# Synthesis and Characterization of Unsymmetrical Double-Decker Siloxane (Basket Cage)

**DOI:** 10.3390/molecules24234252

**Published:** 2019-11-22

**Authors:** Rungthip Kunthom, Nobuhiro Takeda, Masafumi Unno

**Affiliations:** Department of Chemistry and Chemical Biology, Graduate School of Science and Technology, Gunma University, Gunma, Kiryu 376-8515, Japan; t172a002@gunma-u.ac.jp (R.K.); ntakeda@gunma-u.ac.jp (N.T.)

**Keywords:** basket shape, unsymmetrical structure, functional siloxane, all phenyl groups, well-defined compound

## Abstract

The one-pot synthesis of an unsymmetrical double-decker siloxane with a novel structure via the reaction of double-decker tetrasodiumsilanolate with 1 equiv. of dichlorotetraphenyldisiloxane in the presence of an acid is reported herein for the first time. The target compound bearing all phenyl substituents on the unsymmetrical siloxane structure was successfully obtained, as confirmed by ^1^H-NMR, ^13^C-NMR, ^29^Si-NMR, IR, MALDI-TOF, and X-ray crystallography analyses. Additionally, the thermal properties of the product were evaluated by TG/DTA and compared with those of other siloxane cage compounds.

## 1. Introduction

Many scientists have been interested in silsesquioxane cage materials with a well-defined structure because such materials have a low dielectric constant and excellent thermal stability [1,2,3,4,5,6,7]. Additionally, these compounds find a wide range of applications in polymers [8,9,10,11], sensors [12], catalysis [7,8,9,13], organic light-emitting diodes [14], host-guest complexes [15,16], and porous materials [17]. Most of the double-decker silsesquioxanes (DDSQ) [10,11,14,18,19,20,21,22,23,24,25,26] and polyhedral oligomeric silsesquioxanes [7,8,9,12,13,15,16,17,27,28,29] have a symmetrical structure with the same organic functional group on both sides. In contrast, only a few studies on unsymmetrical silsesquioxane or siloxane cages have been reported because of the difficulty in obtaining a perfect Janus structure via a simple hydrolytic condensation of two organomonosilane precursors. Due to its unique chemical/physical properties related to type, number, and position of organic functional groups on a single molecule, the synthesis of asymmetrical structures has recently become an essential subject in chemistry [30,31,32,33,34,35]. Since the first report by the Laine group [30], to the most recent by the Kuroda group [31], on the synthesis of Janus cube octasilsesquioxane by the hydrolytic condensation of trimethoxy groups on cyclotetrasiloxanes rings, only our group has successfully synthesized a Janus cube from two different organic functional groups on cyclotetrasiloxane precursors [32]. Furthermore, we also expanded to the synthesizing of an elongated core-sized Lantern Cage siloxane with the similar capping reaction between tetrakis(bromodimethylsiloxy)tetraphenylcyclotetrasiloxane and cyclotetrasiloxanetetraol [33].

Recently, the Maleczka Jr. and Lee group obtained an unsymmetrical DDSQ with the introduction of a trichloromethylsilane on one side of the DDSQ. This caused difficulty in controlling the synthesis, and purification required a vast difference in polarity between the product and the undesired compound [34]. One year later, this group succeeded in synthesizing an unsymmetrical DDSQ by using *p*-MeO-C_6_H_4_B(OH)_2_ as the protecting group for each side of the reactive silanol group [35]. Developing an effective synthesis procedure for unsymmetrical DDSQ materials remains an interesting topic.

To date, the synthetic pathway of a novel unsymmetrical cage with one-side capping as a model compound for highly regulated silsesquioxanes has not been studied. Meanwhile, a siloxane material bearing phenyl groups has attracted attention for photochemical applications because of its high refractive index and high temperature stability [18,36]. In this work, we prepared a novel well-defined unsymmetrical DDSQ via the formation of DDSQ-ONa with 1 equiv. of dichlorotetraphenyldisiloxane (ClPh_2_Si)_2_O for the first time. This method allows for control of the unsymmetrical closed-cage DDSQ structure with a disiloxane substituent, in addition to being simple and inexpensive; moreover, the reaction proceeds to completion in a short time, with the chloride ligand being an excellent leaving group.

## 2. Results and Discussion

**Scheme 1:**
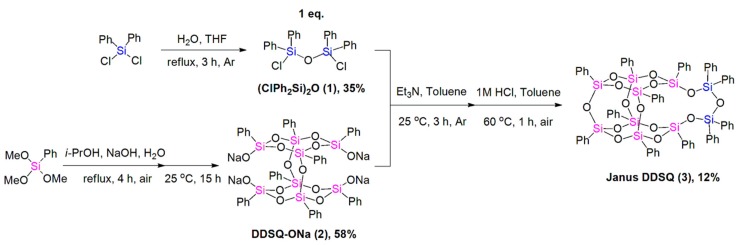
Synthesis of unsymmetrical double-decker silsesquioxanes (DDSQ) (**3**)**.**

Dichlorodisiloxane **1** [37] was freshly prepared by the hydrolytic condensation of dichlorodiphenylsilane in the presence of water for 3 h. The product obtained after distillation was a viscous colorless oil in 35% yield. The synthesis procedure for DDSQ-ONa (**2**) is described in detail in previous reports [19,21,38]. A 2-propanol solution of trimethoxyphenylsilane with NaOH and water was refluxed for 4 h, followed by continuous stirring at room temperature for 15 h. After filtration, a white solid was obtained in 58% yield. Afterward, unsymmetrical DDSQ (**3**) was synthesized by a capping reaction between **2** and **1** to obtain the closed DDSQ T_8_D_2_-Ph structure, as shown in Scheme 1. Compound **1** was slowly added to the reaction mixture of **2** in 1:1 molar ratio and stirred at room temperature to obtain the expected intermediate, one-side capped DDSQ, with the residual reactive siloxanolate group (Si-O^−^). After 3 h, an acid was directly added to the reaction mixture to induce a one-pot reaction, and hydrolytic condensation led to the formation of **3** since heating at 60 °C for 1 h. Subsequent to the extraction and drying of the crude product, a significant challenge in product purification was isolation from several unwanted byproducts. Compound **3** was isolated as a white solid in 15% yield by column chromatography (CHCl_3_/hexane: 4:6; R_f_ = 0.41). The desired compound was not moisture-sensitive and could be recrystallized by slow evaporation from a CH_2_Cl_2_/2-propanol (1:1) solvent mixture to obtain colorless crystals in 12% yield. The structure of the compound was determined based on spectroscopic data and X-ray crystallography.

**Figure 1:**
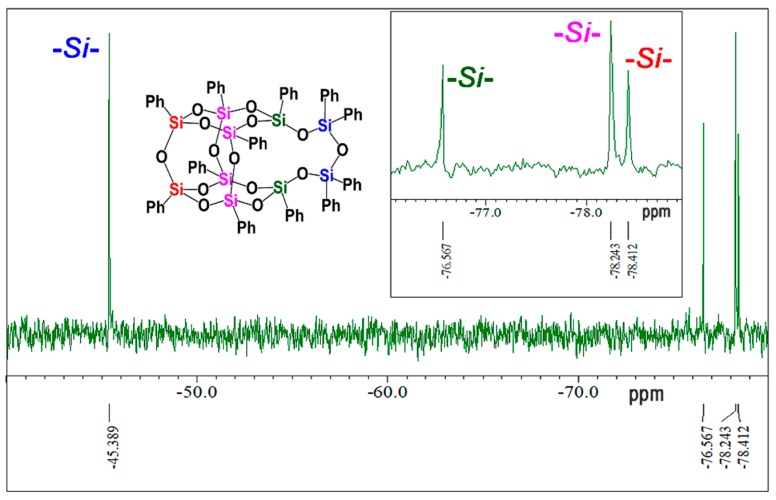
^29^Si{^1^H}-NMR (119.2 MHz, CDCl_3_) spectrum of **3** with labeled silicon positions.

The ^1^NMR spectrum of **3** in CDCl_3_ (Figure S1) supports the proposed structure of **3** with proton resonances around 7.13–7.60 ppm attributed to the phenyl groups. Spectra of ^13^C (Figure S2) and ^13^C DEPT-90 (Figure S3) NMR showed resonances for the phenyl carbons at 127.5–134.5 ppm. The ^29^Si{^1^H}-NMR spectrum of **3** (Figure 1) showed four singlet signals at −45.4, −76.6, −78.2, and −78.4 ppm assignable to four types of silicon atoms in a cage. Silicon resonance signals at −76.6, −78.2, and −78.4 ppm, which were derived from DDSQ, were shifted significantly upfield relative to the T type silicon, while the introduction of tetraphenyldisiloxane showed a downfield-shifted signal at −45.4 ppm [18,37]. The ^29^Si-NMR chemical shifts of related compounds are summarized in Table 1.

The IR spectrum of **3** was shown in Figure S6. Strong Si-O-Si stretching absorption [18] at 1090 and 1113 cm^−1^ confirmed that the DDSQ core consisted of disiloxane, which was unperturbed even after acid addition during the synthesis. Meanwhile, notable absorption bands appeared at 3007–3072 cm^−1^ (Ar-H) and 1134 cm^−1^ (Si-Ph). Additionally, the structure of 3 was supported by the MALDI-TOF mass spectrum (Figure S5), which showed molecular ions at *m/z* 1452.48 g mol^−1^ [M + Na]^+^, calculated value 1452.16.

**Figure 2:**
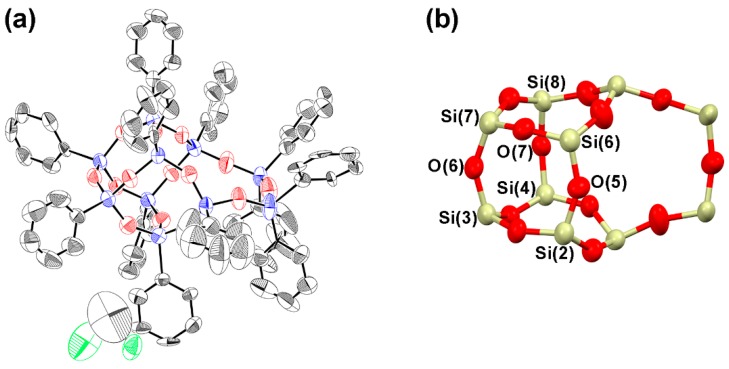
X-ray crystallography data. (**a**) ORTEP (Oak Ridge Thermal- Ellipsoid Plot Program) diagram of **3** in perspective view. Ellipsoids are drawn at the 30% probability level. Hydrogen atoms are omitted for clarity. Black: carbon; blue: silicon; red: oxygen; green: chlorine. (**b**) Side view of **3** with number labels. Hydrogen atoms and phenyl rings are omitted for clarity. Yellow: silicon; red: oxygen.

The crystal structure (Figure 2) from X-ray crystallography analysis strongly supported the formation of **3**. There are two different siloxane bonds (Si-O-Si) that differ in their positions linked to two decks of tetracyclic siloxane: 1) Si(2)-O(5)-Si(6) and Si(4)-O(7)-Si(8), which connect the pairs of cyclotetrasiloxane decks in the middle; and 2) Si(3)-O(6)-Si(7), which join at the end positions. The average Si-Si distances are 3.220(3) and 3.237(6) Å, which fall within the range commonly found in siloxanes. Moreover, the average Si-O-Si angle in 3 is about 144.9(4)–165.2(5) Å, which is larger than that of T_8_-Ph (149(5) Å) [40] and symmetrical double-decker silsesquioxanes (140.1(1)–165.2(1) Å) [18] due to the constraints imposed by the ring structure. In these three cases, the Si-O-Si bonds are linked to form a six-faced well-defined structure, but compound **3** forms a structure with eight- and twelve-membered rings. These results show that bond expansion occurred due to the disiloxane linkage. The resulting structure is similar to a basket in the view of DDSQ as the core and the flexible disiloxane as the handle.

**Figure 3:**
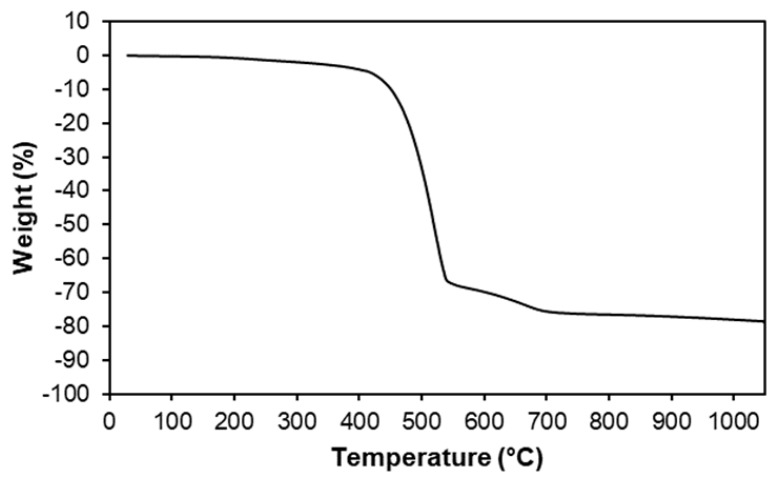
TG/DTA results obtained under nitrogen atmosphere. Heating rate is 10 °C/min.

Thermogravimetric analysis (TGA) of 3 (Figure 3) was performed under air at a heating rate of 10 °C/min, over the temperature range of 30 °C to 1000 °C. The Td_5_ (5% weight loss) values in Table 2 show that this material has high thermal stability up to 417 °C, but its stability is significantly lower than that of the octaphenylsilsesquioxane (T_8_-Ph) with a symmetrical cage structure and di(diphenylsilyl)-bridged double-decker octaphenylsilsesquioxane (Sym-T_8_D_2_-Ph) because of the loose structural packing. At a high temperature of 1000 °C, a small amount of residual silica is left; about 22%, was observed. It is noteworthy that compound 3 showed similar thermal stability to T_8_-Ph even containing d-siloxane moiety. This fact also indicates that 3 is the promising precursor of thermally-stable well-defined materials.

## 3. Materials and Methods 

### 3.1. Materials and Instruments

Reaction solvents: toluene, 2-propanol, and tetrahydrofuran (THF) were purchased from FUJIFILM Wako Chemical Industry, Ltd. (Osaka, Japan). Pure phenyltrimethoxysilane and dichlorodiphenylsilane were purchased from Tokyo Chemical Industry Co., Ltd. (Tokyo, Japan) and used without further purification. Sodium hydroxide was purchased from Kanto Chemical Co. Inc. (Tokyo, Japan). Triethylamine was distilled and stored over potassium hydroxide under argon atmosphere, with protection from light. Toluene was dried over calcium hydride (CaH_2_) and stored under argon atmosphere with 4 Å activated molecular sieves until use. 

Fourier transform nuclear magnetic resonance (NMR) spectra were recorded on JEOL JNM-ECA (Tokyo, Japan) 400 (^1^H at 399.78 MHz) and JEOL JNM-ECS 600 (^13^C at 150.91 MHz and ^29^Si at 119.24 MHz) instruments, in CDCl_3_ using SiMe_4_ (TMS) as an internal standard. Chemical shifts were reported as δ units (ppm), and the residual solvent peaks were used as standards. Elemental analyses were performed by the Center for Material Research by Instrumental Analysis (CIA), Gunma University, Japan. MALDI-TOF mass analysis was carried out with a Shimadzu AXIMA Performance instrument (Kyoto, Japan) using 2,5-dihydroxybenzoic acid (dithranol) as the matrix and AgNO_3_ as the ion source. Infrared spectra were obtained using a Shimadzu FTIR-8400S instrument. TGA analyses were performed on a Rigaku thermal gravimetric analyzer (Thermoplus TG-8120, Tokyo, Japan). All samples were heated from 30 °C to 1000 °C at a heating rate of 10 °C min^−1^, under N_2_. The crystal data were collected on a Rigaku XtaLab P200 diffractometer with multi-layer mirror monochromated Mo K_α_ radiation (λ = 0.71075 Å). The structures were solved by direct methods (SHELXS-974), and refined by full-matrix least-squares procedures on F2 for all reflections (SHELXL-97). All the non-hydrogen atoms were refined anisotropically. All hydrogens were positioned using AFIX instructions. All calculations were carried out using Yadokari-XG2009 (Sendai, Japan).

### 3.2. Synthesis of Unsymmetrical T_8_D_2_, ***3***

1,3-Dichloro-1,1,3,3-diphenyldisiloxane ((ClPh_2_Si)_2_O, **1**) and tetrasodiooctaphenyltetracyclo- octasilsesquioxanolate (DDSQ-ONa, **2**) were freshly prepared according to reported procedures [21,37]. Compound **1** (0.15 mL, 0.86 mmol) was added dropwise to a solution of (DDSQ-ONa, **2**) (1.0 g, 0.86 mmol), triethylamine (0.24 mL, 1.72 mmol), and dried toluene (10 mL) over 15 min at 0 °C under argon atmosphere. Stirring was further continued for 3 h at room temperature. Subsequently, 1 M HCl (aq) (2 mL) in toluene (20 mL) was added to this solution and stirring was continued for 1 h at 60 °C. Then, the organic layer was washed with water (5 mL), aqueous sat. NaHCO_3_ (5 mL), and water (10 mL). The organic layer was dried over anhydrous Na_2_SO_4_, filtered, and evaporated. The crude product was obtained as the mixture between the white solid of the target compound and the viscous liquid of undesired byproduct. The target product (R_f_ = 0.41) was isolated by column chromatography (chloroform/hexane (4:6)) and recrystallized from a mixture of dichloromethane and 2-propanol (1:1) to obtain colorless crystals (0.15 g; 12% yield).

Spectral data for 3: ^1^H-NMR (400 MHz, CDCl_3_): δ 7.11–7.19 (m, 20H), 7.26–7.46 (m, 24H), 7.49–7.51 (m, 4H), 7.58–7.60 (m, 8H), 7.69–7.71 (m, 4H). ^13^C-NMR (150.9 MHz, CDCl_3_): δ 127.46 (CH), 127.56 (CH), 127.70 (CH), 127.85 (CH), 129.88 (CH), 130.23 (CH), 130.31 (CH), 130.40 (C), 130.70 (CH), 131.98 (C), 133.97 (CH), 134.09 (CH, C; overlapped), 134.14 (CH), 134.43 (C), 134.48 (CH) ppm. ^29^Si-NMR (119.2 MHz, CDCl_3_): δ −45.4, −76.6, −78.2, −78.4 ppm. IR (KBr): 494, 521, 696, 729, 741, 999, 1028, 1090, 1113, 1134, 1431, 1595, 3007, 3049, 3072 cm^−1^. MALDI-TOF MS (*m*/*z*): 1452.48 ([M + Na]^+^, calcd. 1452.16).

## 4. Conclusions

Tetraphenylsiloxyl-bridged double-decker octaphenylsilsesquioxane (**3**), an unsymmetrical DDSQ, was successfully synthesized starting from DDSQ-ONa under mild conditions and characterized using various techniques. The Td_5_ value of **3** at 417 °C indicated its high thermal stability. Because of its unique structure within all-phenyl groups, this compound may be advantageous for the encapsulation of ions and photochemical applications. Additionally, this simple synthesis protocol would be useful as a prototype for the preparation of organofunctional siloxane compounds. The disiloxane precursor with other reactive and/or unreactive organic functional groups could be introduced in the future.

## Figures and Tables

**Table 1:** ^29^Si{^1^H}-NMR data of all-phenyl siloxane compounds.

Compound	Si Unit Type	Signal (ppm)
T_8_-Ph [39]	T	−78.4
T_12_-Ph [40]	T	−77.0, −80.6
*sym*-DDSQ-Ph (T_8_D_2_) [18,38]	T, D	−45.4, −78.1, −79.4
(ClPh_2_Si)_2_O [37]	D	−18.6
3	T, D	−45.4, −76.6, −78.2, −78.4

**Table 2:** TG/DTA results obtained under nitrogen atmosphere.

Compound	Si+O Ratio (%)	Td_5_ (°C)	Residue at 1000 °C (%)
T_8_-Ph [18]	40	439	50
*sym*-DDSQ-Ph (T_8_D_2_) [18]	35	425	N/A
**3**	35	417	22

## Supplementary Materials

Additional characterization data are available as electronic supporting information: Figure S1 ^1^H-NMR (400 MHz, CDCl_3_) spectrum of **3**; Figure S2 ^13^C{^1^H}-NMR (150.9 MHz, CDCl_3_) spectrum of **3**; Figure S3 ^13^C-DEPT-90 NMR (150.9 MHz, CDCl_3_) spectrum of **3**; Figure S4 ^29^Si-{^1^H} NMR (119.2 MHz, CDCl_3_) spectrum of **3**; Figure S5 MALDI-TOF MS spectrum of **3**; Figure S6 IR spectrum of **3**; Figure S7 TG/DTA thermogram of **3** at a heating rate of 10 °C/min under nitrogen; Figure S8 ORTEP diagram of **3** with atomic and number labels; Figure S9 Packing diagram for 3; Table S1 Crystal data and structure refinement for **3**; Table S2 Bond lengths [Å] and bond angles [°] for **3**.
