# Supplementary material for: Synthesis and Characterization of Unsymmetrical Double-Decker Siloxane (Basket Cage)
Source: Molecules. 2019 Nov 22;24(23):4252. doi: 10.3390/molecules24234252 (PMC6930635; doi:10.3390/molecules24234252)
Supplement: Supplementary file 1 [file molecules-24-04252-s001.pdf]

Supporting information

Synthesis and Characterization of Unsymmetrical Double-decker siloxane (Basket Cage)

Rungthip Kunthom, Nobuhiro Takeda, Masafumi Unno*

Department of Chemistry and Chemical Biology, Graduate School of Science and Technology, Gunma University, Kiryu 376-8515, Gunma, Japan

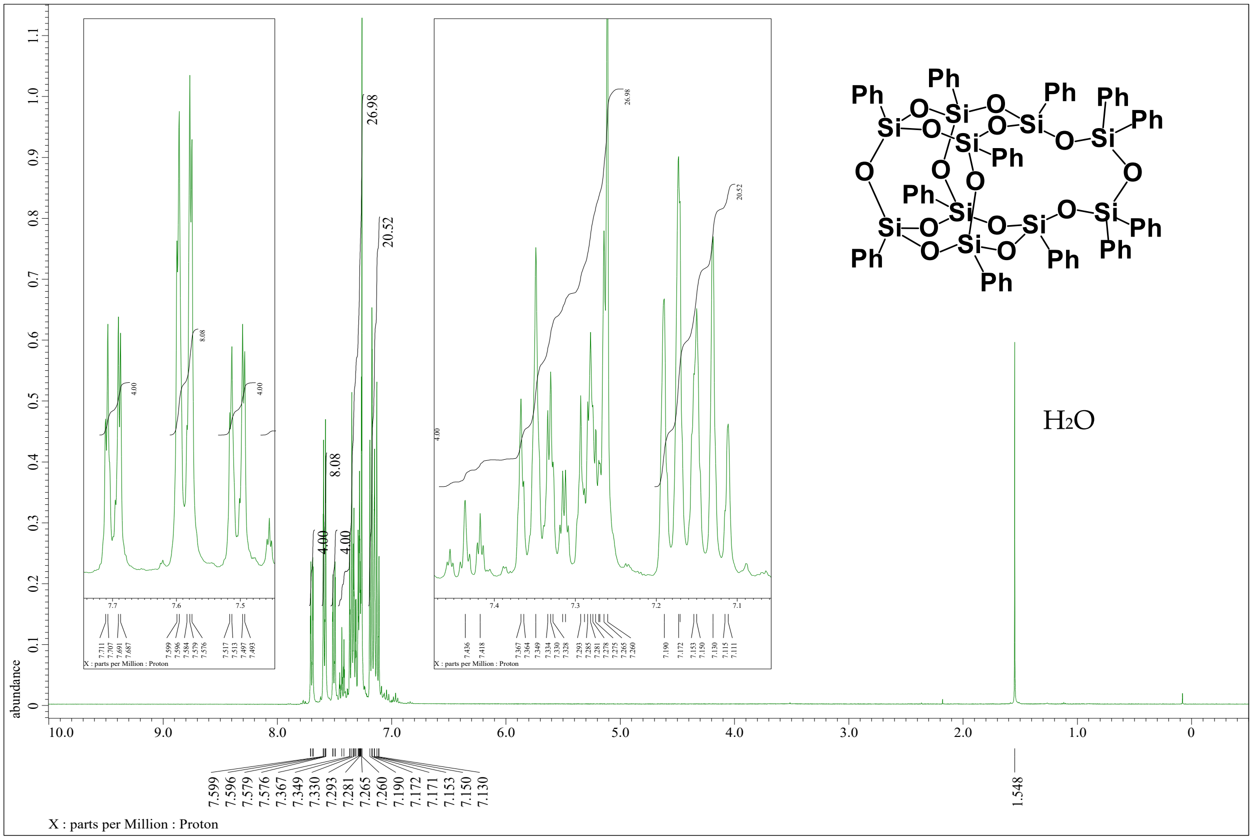

Figure S1 ^{1}H NMR (400 MHz, $CDCl_3$) spectrum of **3**

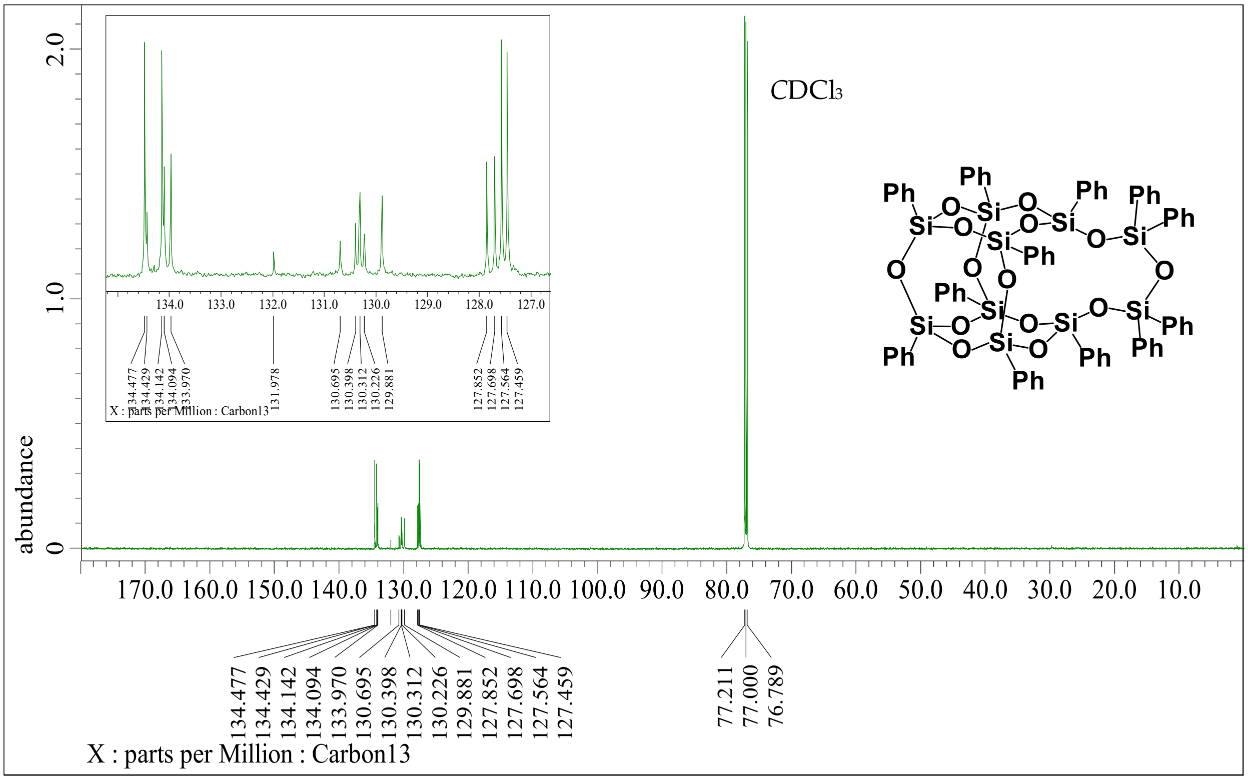

Figure S2 $^{13}C\{^1H\}$ NMR (150.9 MHz, $CDCl_3$) spectrum of **3**

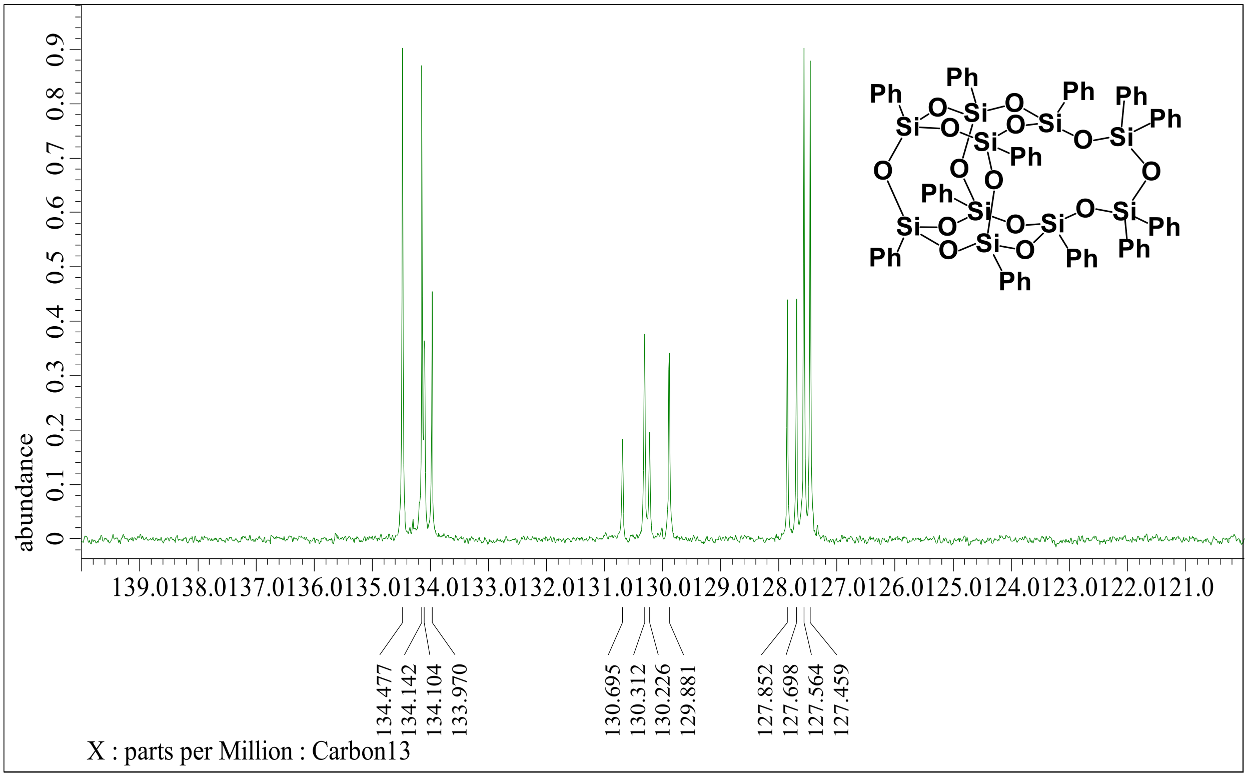

Figure S3 ^{13}C DEPT-90 NMR (150.9 MHz, $CDCl_3$) spectrum of **3**

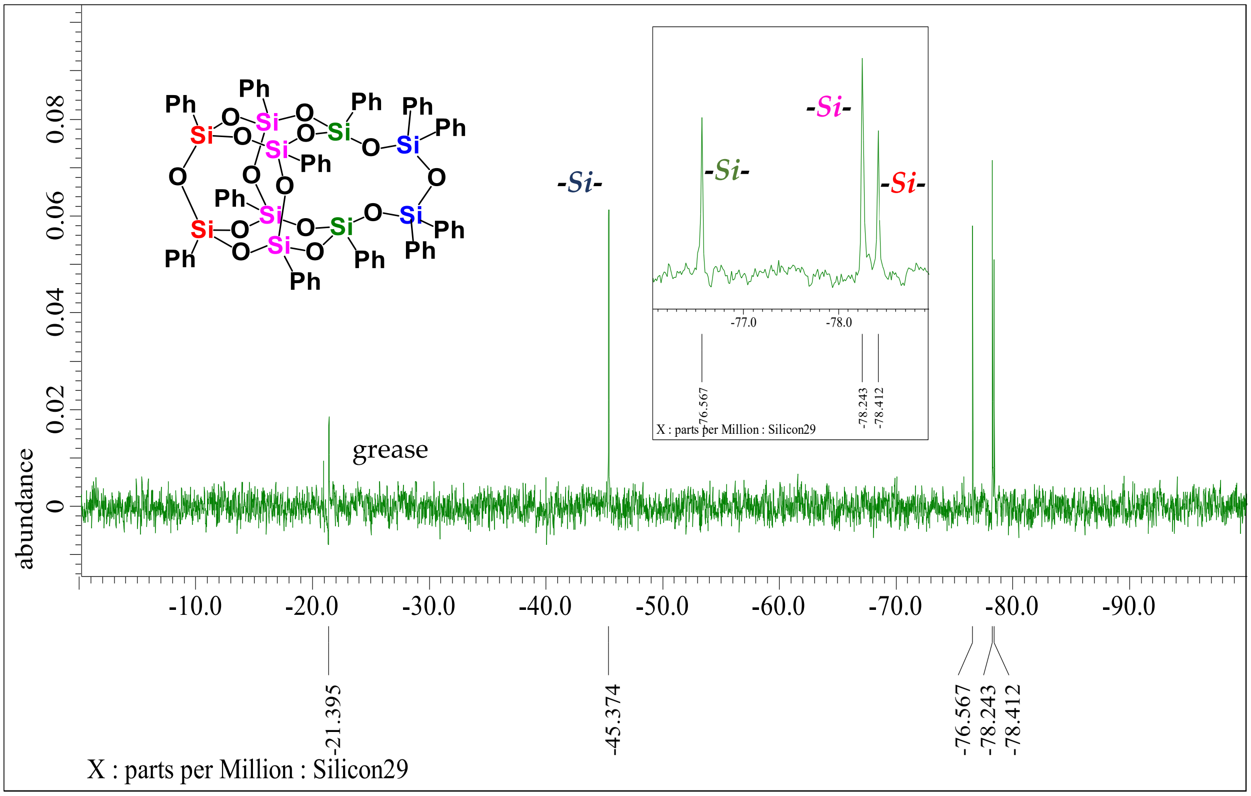

Figure S4 $^{29}Si\{^1H\}$ NMR (119.2 MHz, $CDCl_3$) spectrum of **3**

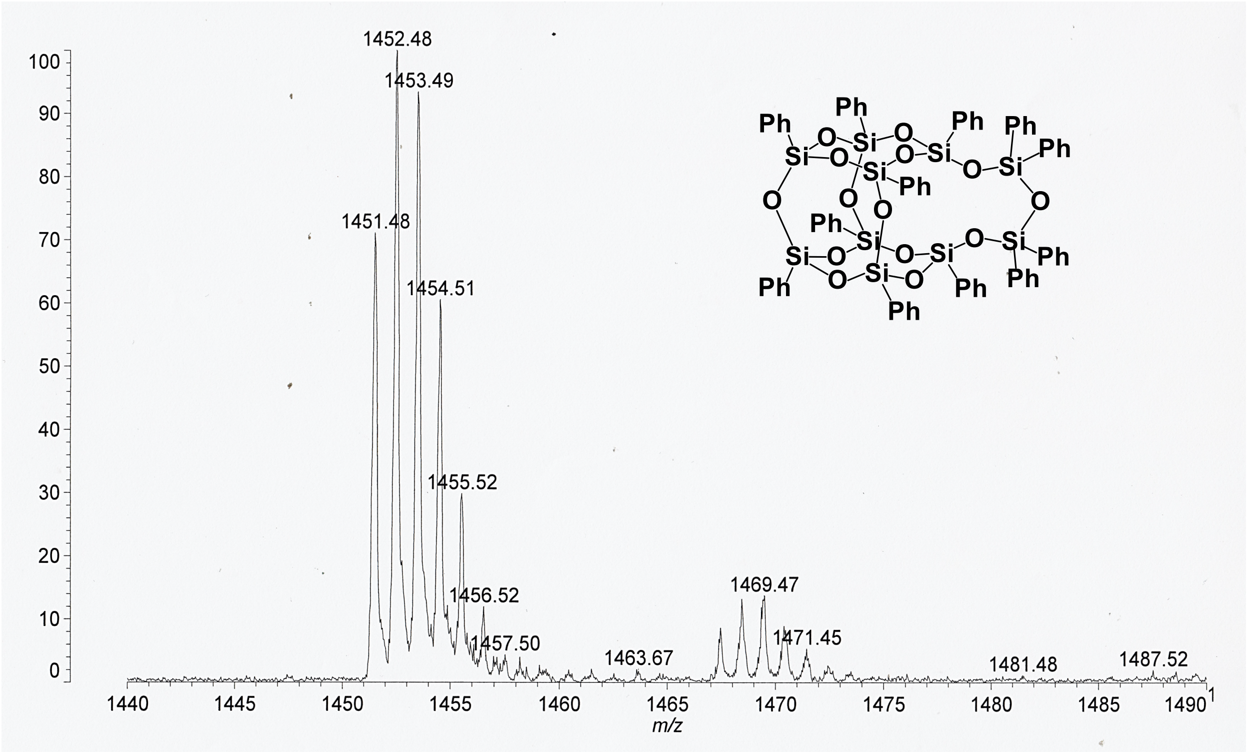

Figure S5 MALDI-TOF MS spectrum of **3**

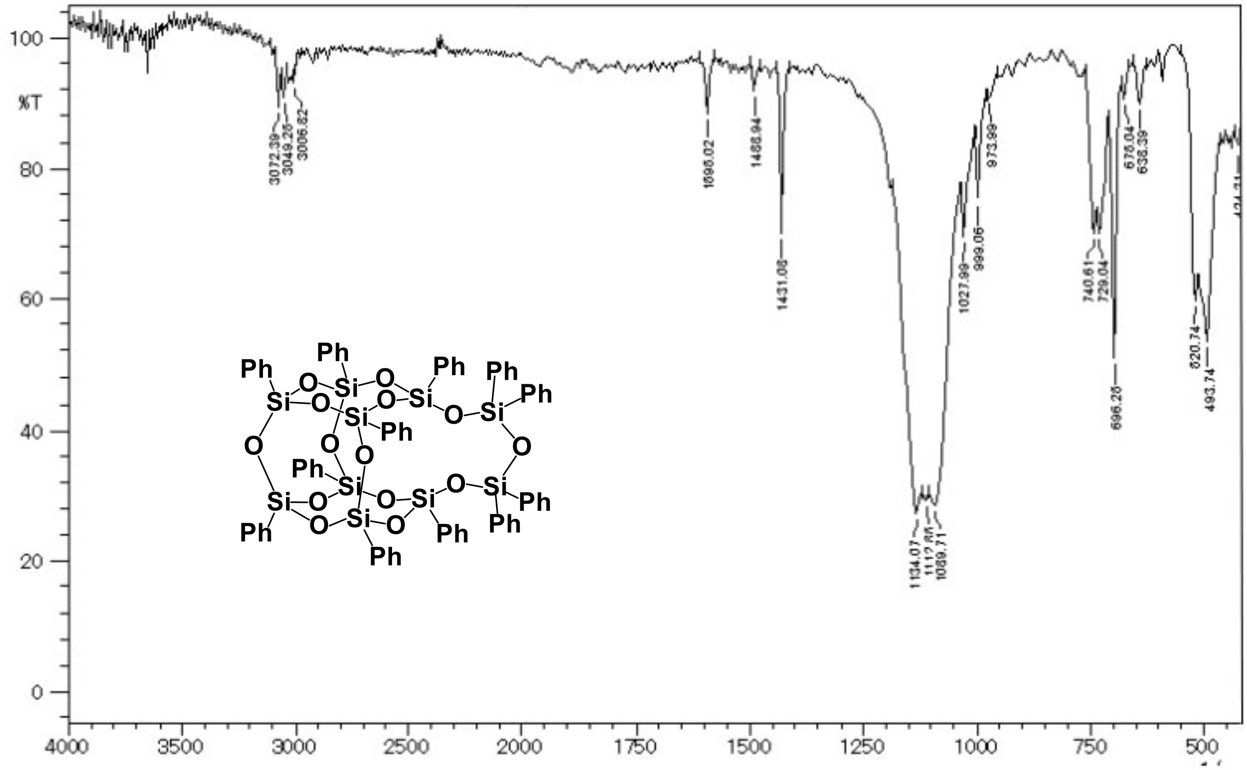

Figure S6 IR spectrum of **3**

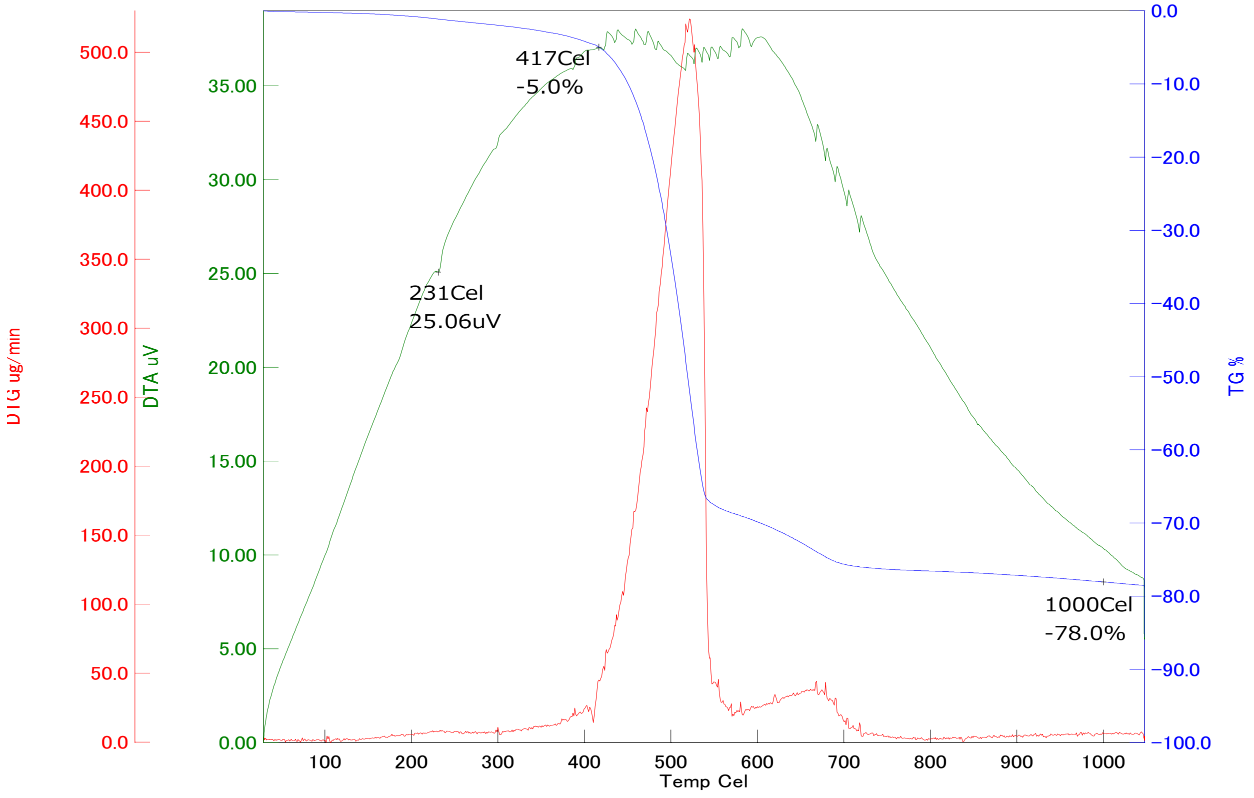

Figure S7 TG/DTA thermogram of **3** with heating rate is 10 °C/min under nitrogen

X-ray Crystallographic Analysis of 3

Crystallographic data had been deposited with Cambridge Crystallographic Data Centre: Deposition numbers CCDC 1966252 for compound **3**. Copies of the data can be obtained free of charge via http://www.ccdc.cam.ac.uk/conts/retrieving.html (or from the Cambridge Crystallographic Data Centre, 12, Union Road, Cambridge, CB2 1EZ, U.K.; Fax: +44 1223 336033; e-mail: deposit@ccdc.cam.ac.uk).

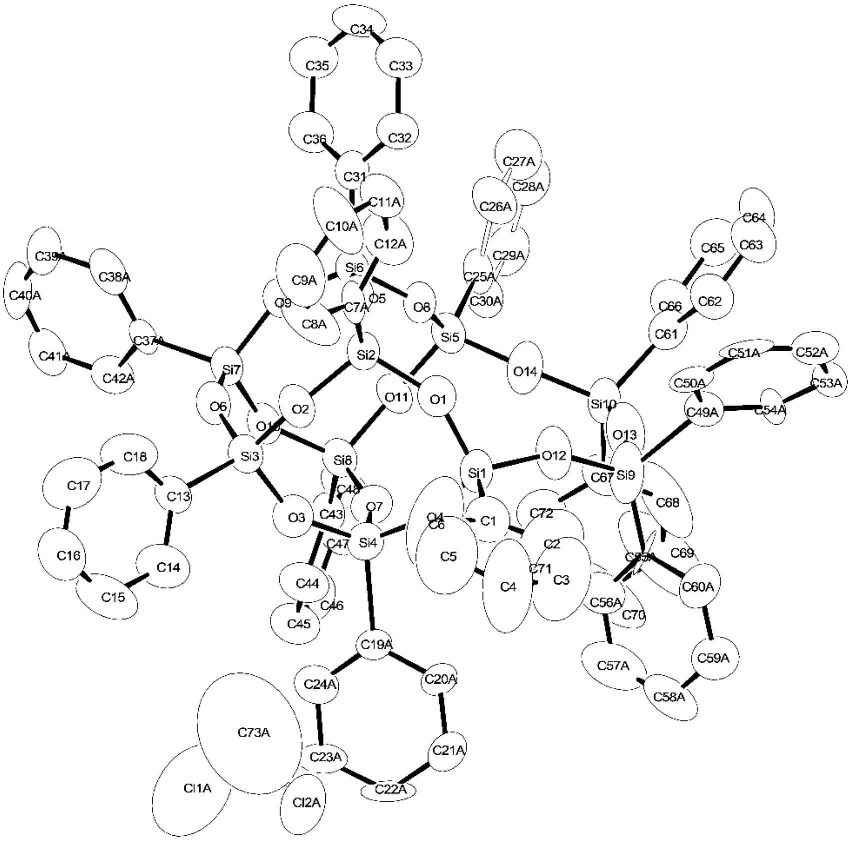

Figure S8 ORTEP diagram of **3** with number label.

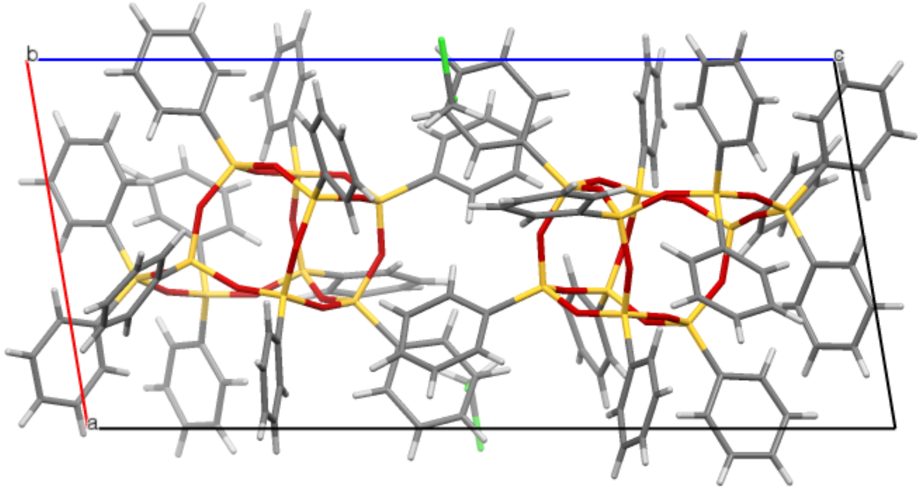

Figure S9 Packing diagram for **3**.

Table S1 Crystal data and structure refinement for 3.

Empirical formula	C145 H122 Cl2 O28 Si20	
Formula weight	1472.56	
Temperature	130 K	
Wavelength	0.71075 Å	
Crystal system	Triclinic	
Space group	P-1	
Unit cell dimensions	a = 11.113(5) Å	α = 88.429(17)°.
	b = 14.053(7) Å	β = 80.435(15)°.
	c = 23.828(12) Å	γ = 82.488(17)°.
Volume	3638(3) $Å^3$	
Z	2	
Density (calculated)	1.344 Mg/m^3	
Absorption coefficient	0.281 mm^{-1}	
F(000)	1530	
Crystal size	0.17 x 0.02 x 0.01 mm^3	
Theta range for data collection	2.520 to 27.494°.	
Index ranges	-13<=h<=14, -18<=k<=17, -30<=l<=30	
Reflections collected	59700	
Independent reflections	16541 [R(int) = 0.2892]	
Completeness to theta = 25.242°	99.6 %	
Absorption correction	Numerical	
Max. and min. transmission	1.000 and 0.203	
Refinement method	Full-matrix least-squares on F^2	
Data / restraints / parameters	16541 / 385 / 1287	
Goodness-of-fit on F^2	0.994	
Final R indices [I>2sigma(I)]	R1 = 0.1076, wR2 = 0.2107	
R indices (all data)	R1 = 0.3367, wR2 = 0.3258	
Extinction coefficient	n/a	
Largest diff. peak and hole	0.457 and -0.340 e.$Å^{-3}$	

Table S2. Bond lengths [Å] and angles [°] for **3**.

Si(1)-O(12)	1.594(6)	Si(2)-O(5)	1.613(6)
Si(1)-O(4)	1.602(7)	Si(2)-O(2)	1.615(6)
Si(1)-O(1)	1.604(7)	Si(2)-C(7B)	1.74(4)
Si(1)-C(1)	1.853(12)	Si(2)-C(7A)	1.88(2)
O(1)-Si(2)	1.625(7)	O(2)-Si(3)	1.622(6)

Si(3)-O(3)	1.617(7)	O(13)-Si(10)	1.606(7)
Si(3)-O(6)	1.620(7)	Si(10)-O(14)	1.610(7)
Si(3)-C(13)	1.841(9)	Si(10)-C(61)	1.841(10)
O(3)-Si(4)	1.609(6)	Si(10)-C(67)	1.856(10)
Si(4)-O(7)	1.600(6)	C(1)-C(2)	1.346(17)
Si(4)-O(4)	1.610(7)	C(1)-C(6)	1.349(17)
Si(4)-C(19B)	1.73(4)	C(2)-C(3)	1.40(2)
Si(4)-C(19C)	1.81(3)	C(2)-H(1)	0.9500
Si(4)-C(19A)	1.96(2)	C(3)-C(4)	1.29(2)
O(5)-Si(6)	1.605(6)	C(3)-H(2)	0.9500
O(6)-Si(7)	1.617(6)	C(4)-C(5)	1.28(2)
O(7)-Si(8)	1.623(6)	C(4)-H(3)	0.9500
Si(5)-O(14)	1.595(7)	C(5)-C(6)	1.50(2)
Si(5)-O(8)	1.611(6)	C(5)-H(4)	0.9500
Si(5)-O(11)	1.620(6)	C(6)-H(5)	0.9500
Si(5)-C(25A)	1.82(2)	C(7A)-C(8A)	1.34(3)
Si(5)-C(25B)	1.883(17)	C(7A)-C(12A)	1.37(3)
O(8)-Si(6)	1.610(6)	C(8A)-C(9A)	1.41(2)
Si(6)-O(9)	1.628(6)	C(8A)-H(31)	0.9500
Si(6)-C(31)	1.830(10)	C(9A)-C(10A)	1.25(3)
O(9)-Si(7)	1.613(6)	C(9A)-H(32)	0.9500
Si(7)-O(10)	1.624(6)	C(10A)-C(11A)	1.30(3)
Si(7)-C(37B)	1.80(4)	C(10A)-H(33)	0.9500
Si(7)-C(37A)	1.833(16)	C(11A)-C(12A)	1.48(3)
O(10)-Si(8)	1.623(6)	C(11A)-H(34)	0.9500
Si(8)-O(11)	1.621(6)	C(12A)-H(35)	0.9500
Si(8)-C(43)	1.842(10)	C(7B)-C(12B)	1.385(19)
O(12)-Si(9)	1.634(6)	C(7B)-C(8B)	1.386(19)
Si(9)-C(55A)	1.54(3)	C(8B)-C(9B)	1.391(19)
Si(9)-O(13)	1.618(8)	C(8B)-H(41)	0.9500
Si(9)-C(49B)	1.76(2)	C(9B)-C(10B)	1.385(19)
Si(9)-C(55B)	2.03(3)	C(9B)-H(42)	0.9500
Si(9)-C(49A)	2.09(4)	C(10B)-C(11B)	1.392(19)

C(10B)-H(43)	0.9500	C(21B)-H(52)	0.9500
C(11B)-C(12B)	1.410(19)	C(22B)-C(23B)	1.394(19)
C(11B)-H(44)	0.9500	C(22B)-H(53)	0.9500
C(12B)-H(45)	0.9500	C(23B)-C(24B)	1.400(19)
C(13)-C(14)	1.357(13)	C(23B)-H(54)	0.9500
C(13)-C(18)	1.375(15)	C(24B)-H(55)	0.9500
C(14)-C(15)	1.400(15)	C(19C)-C(24C)	1.394(19)
C(14)-H(6)	0.9500	C(19C)-C(20C)	1.406(18)
C(15)-C(16)	1.336(16)	C(20C)-C(21C)	1.400(19)
C(15)-H(7)	0.9500	C(20C)-H(56)	0.9500
C(16)-C(17)	1.336(17)	C(21C)-C(22C)	1.389(19)
C(16)-H(8)	0.9500	C(21C)-H(57)	0.9500
C(17)-C(18)	1.399(16)	C(22C)-C(23C)	1.380(18)
C(17)-H(9)	0.9500	C(22C)-H(58)	0.9500
C(18)-H(10)	0.9500	C(23C)-C(24C)	1.391(18)
C(19A)-C(24A)	1.394(18)	C(23C)-H(59)	0.9500
C(19A)-C(20A)	1.398(18)	C(24C)-H(60)	0.9500
C(20A)-C(21A)	1.376(17)	C(25A)-C(30A)	1.390(18)
C(20A)-H(46)	0.9500	C(25A)-C(26A)	1.392(19)
C(21A)-C(22A)	1.390(18)	C(26A)-C(27A)	1.398(19)
C(21A)-H(47)	0.9500	C(26A)-H(91)	0.9500
C(22A)-C(23A)	1.397(18)	C(27A)-C(28A)	1.392(19)
C(22A)-H(48)	0.9500	C(27A)-H(92)	0.9500
C(23A)-C(24A)	1.396(18)	C(28A)-C(29A)	1.388(18)
C(23A)-H(49)	0.9500	C(28A)-H(93)	0.9500
C(24A)-H(50)	0.9500	C(29A)-C(30A)	1.398(17)
C(19B)-C(24B)	1.391(19)	C(29A)-H(94)	0.9500
C(19B)-C(20B)	1.397(19)	C(30A)-H(95)	0.9500
C(20B)-C(21B)	1.390(18)	C(25B)-C(30B)	1.39(2)
C(20B)-Cl(2B)	1.87(4)	C(25B)-C(26B)	1.42(3)
C(20B)-H(51)	0.9500	C(26B)-C(27B)	1.40(3)
C(21B)-C(22B)	1.382(19)	C(26B)-H(86)	0.9500
C(21B)-Cl(2B)	2.14(4)	C(27B)-C(28B)	1.33(4)

C(27B)-H(87)	0.9500	C(39B)-H(82)	0.9500
C(28B)-C(29B)	1.36(3)	C(40B)-C(41B)	1.391(19)
C(28B)-H(88)	0.9500	C(40B)-H(83)	0.9500
C(29B)-C(30B)	1.37(2)	C(41B)-C(42B)	1.401(19)
C(29B)-H(89)	0.9500	C(41B)-H(84)	0.9500
C(30B)-H(90)	0.9500	C(42B)-H(85)	0.9500
C(31)-C(36)	1.372(13)	C(43)-C(48)	1.381(13)
C(31)-C(32)	1.399(13)	C(43)-C(44)	1.385(13)
C(32)-C(33)	1.367(14)	C(44)-C(45)	1.385(15)
C(32)-H(11)	0.9500	C(44)-H(16)	0.9500
C(33)-C(34)	1.342(16)	C(45)-C(46)	1.364(15)
C(33)-H(12)	0.9500	C(45)-H(17)	0.9500
C(34)-C(35)	1.360(17)	C(46)-C(47)	1.387(16)
C(34)-H(13)	0.9500	C(46)-H(18)	0.9500
C(35)-C(36)	1.395(15)	C(47)-C(48)	1.356(14)
C(35)-H(14)	0.9500	C(47)-H(19)	0.9500
C(36)-H(15)	0.9500	C(48)-H(20)	0.9500
C(37A)-C(42A)	1.38(2)	C(49A)-C(54A)	1.386(19)
C(37A)-C(38A)	1.44(3)	C(49A)-C(50A)	1.39(2)
C(38A)-C(39A)	1.40(2)	C(50A)-C(51A)	1.390(19)
C(38A)-H(36)	0.9500	C(50A)-H(61)	0.9500
C(39A)-C(40A)	1.38(3)	C(51A)-C(52A)	1.392(19)
C(39A)-H(37)	0.9500	C(51A)-H(62)	0.9500
C(40A)-C(41A)	1.33(3)	C(52A)-C(53A)	1.393(19)
C(40A)-H(38)	0.9500	C(52A)-H(63)	0.9500
C(41A)-C(42A)	1.40(2)	C(53A)-C(54A)	1.390(18)
C(41A)-H(39)	0.9500	C(53A)-H(64)	0.9500
C(42A)-H(40)	0.9500	C(54A)-H(65)	0.9500
C(37B)-C(38B)	1.388(19)	C(49B)-C(50B)	1.35(3)
C(37B)-C(42B)	1.394(19)	C(49B)-C(54B)	1.39(3)
C(38B)-C(39B)	1.394(18)	C(50B)-C(51B)	1.44(3)
C(38B)-H(81)	0.9500	C(50B)-H(66)	0.9500
C(39B)-C(40B)	1.391(19)	C(51B)-C(52B)	1.31(3)

C(51B)-H(67)	0.9500	C(63)-H(22)	0.9500
C(52B)-C(53B)	1.35(3)	C(64)-C(65)	1.402(18)
C(52B)-H(68)	0.9500	C(64)-H(23)	0.9500
C(53B)-C(54B)	1.39(2)	C(65)-C(66)	1.408(15)
C(53B)-H(69)	0.9500	C(65)-H(24)	0.9500
C(54B)-H(70)	0.9500	C(66)-H(25)	0.9500
C(55A)-C(60A)	1.378(18)	C(67)-C(68)	1.346(15)
C(55A)-C(56A)	1.383(19)	C(67)-C(72)	1.385(13)
C(56A)-C(57A)	1.398(18)	C(68)-C(69)	1.38(2)
C(56A)-H(71)	0.9500	C(68)-H(26)	0.9500
C(57A)-C(58A)	1.388(19)	C(69)-C(70)	1.36(2)
C(57A)-H(72)	0.9500	C(69)-H(27)	0.9500
C(58A)-C(59A)	1.396(18)	C(70)-C(71)	1.35(2)
C(58A)-H(73)	0.9500	C(70)-H(28)	0.9500
C(59A)-C(60A)	1.396(17)	C(71)-C(72)	1.353(14)
C(59A)-H(74)	0.9500	C(71)-H(29)	0.9500
C(60A)-H(75)	0.9500	C(72)-H(30)	0.9500
C(55B)-C(60B)	1.35(3)	C(73A)-Cl(2A)	1.83(11)
C(55B)-C(56B)	1.37(3)	C(73A)-Cl(1A)	2.02(6)
C(56B)-C(57B)	1.37(2)	C(73A)-H(96)	0.9900
C(56B)-H(76)	0.9500	C(73A)-H(97)	0.9900
C(57B)-C(58B)	1.41(4)	C(73B)-Cl(2B)	1.71(9)
C(57B)-H(77)	0.9500	C(73B)-Cl(1B)	2.02(6)
C(58B)-C(59B)	1.29(4)	C(73B)-H(98)	0.9900
C(58B)-H(78)	0.9500	C(73B)-H(99)	0.9900
C(59B)-C(60B)	1.41(3)		
C(59B)-H(79)	0.9500	O(12)-Si(1)-O(4)	108.4(4)
C(60B)-H(80)	0.9500	O(12)-Si(1)-O(1)	107.3(4)
C(61)-C(66)	1.365(13)	O(4)-Si(1)-O(1)	109.9(3)
C(61)-C(62)	1.410(13)	O(12)-Si(1)-C(1)	108.3(5)
C(62)-C(63)	1.351(16)	O(4)-Si(1)-C(1)	113.4(5)
C(62)-H(21)	0.9500	O(1)-Si(1)-C(1)	109.3(5)
C(63)-C(64)	1.374(18)	Si(1)-O(1)-Si(2)	149.7(4)

O(5)-Si(2)-O(2)	109.5(3)	O(14)-Si(5)-O(8)	106.5(3)
O(5)-Si(2)-O(1)	108.5(3)	O(14)-Si(5)-O(11)	109.6(4)
O(2)-Si(2)-O(1)	108.4(3)	O(8)-Si(5)-O(11)	109.9(3)
O(5)-Si(2)-C(7B)	105.0(14)	O(14)-Si(5)-C(25A)	96.6(10)
O(2)-Si(2)-C(7B)	119.3(14)	O(8)-Si(5)-C(25A)	120.0(9)
O(1)-Si(2)-C(7B)	105.7(12)	O(11)-Si(5)-C(25A)	112.9(8)
O(5)-Si(2)-C(7A)	109.3(9)	O(14)-Si(5)-C(25B)	118.6(6)
O(2)-Si(2)-C(7A)	105.2(8)	O(8)-Si(5)-C(25B)	105.4(6)
O(1)-Si(2)-C(7A)	115.8(8)	O(11)-Si(5)-C(25B)	106.6(6)
Si(2)-O(2)-Si(3)	145.5(4)	Si(6)-O(8)-Si(5)	145.1(4)
O(3)-Si(3)-O(6)	108.4(3)	O(5)-Si(6)-O(8)	106.8(3)
O(3)-Si(3)-O(2)	109.0(3)	O(5)-Si(6)-O(9)	109.9(3)
O(6)-Si(3)-O(2)	108.9(3)	O(8)-Si(6)-O(9)	108.5(3)
O(3)-Si(3)-C(13)	109.5(4)	O(5)-Si(6)-C(31)	109.9(4)
O(6)-Si(3)-C(13)	110.8(4)	O(8)-Si(6)-C(31)	112.5(4)
O(2)-Si(3)-C(13)	110.1(4)	O(9)-Si(6)-C(31)	109.2(4)
Si(4)-O(3)-Si(3)	150.0(4)	Si(7)-O(9)-Si(6)	145.9(4)
O(7)-Si(4)-O(3)	110.0(3)	O(9)-Si(7)-O(6)	109.2(3)
O(7)-Si(4)-O(4)	107.4(4)	O(9)-Si(7)-O(10)	109.5(3)
O(3)-Si(4)-O(4)	109.6(3)	O(6)-Si(7)-O(10)	109.0(3)
O(7)-Si(4)-C(19B)	118.4(13)	O(9)-Si(7)-C(37B)	112.0(17)
O(3)-Si(4)-C(19B)	108.3(14)	O(6)-Si(7)-C(37B)	101.5(13)
O(4)-Si(4)-C(19B)	102.7(14)	O(10)-Si(7)-C(37B)	115.3(16)
O(7)-Si(4)-C(19C)	111.1(13)	O(9)-Si(7)-C(37A)	109.3(6)
O(3)-Si(4)-C(19C)	101.5(10)	O(6)-Si(7)-C(37A)	112.5(6)
O(4)-Si(4)-C(19C)	117.0(11)	O(10)-Si(7)-C(37A)	107.3(7)
O(7)-Si(4)-C(19A)	105.1(10)	Si(8)-O(10)-Si(7)	144.9(4)
O(3)-Si(4)-C(19A)	115.8(8)	O(11)-Si(8)-O(10)	109.0(3)
O(4)-Si(4)-C(19A)	108.6(9)	O(11)-Si(8)-O(7)	110.0(3)
Si(1)-O(4)-Si(4)	157.1(4)	O(10)-Si(8)-O(7)	108.1(3)
Si(6)-O(5)-Si(2)	156.9(4)	O(11)-Si(8)-C(43)	107.2(4)
Si(7)-O(6)-Si(3)	146.7(4)	O(10)-Si(8)-C(43)	111.2(4)
Si(4)-O(7)-Si(8)	152.3(4)	O(7)-Si(8)-C(43)	111.4(4)

Si(5)-O(11)-Si(8)	146.5(4)	C(4)-C(5)-C(6)	115.3(17)
Si(1)-O(12)-Si(9)	153.1(5)	C(4)-C(5)-H(4)	122.3
C(55A)-Si(9)-O(13)	110.5(13)	C(6)-C(5)-H(4)	122.3
C(55A)-Si(9)-O(12)	117.4(12)	C(1)-C(6)-C(5)	121.7(15)
O(13)-Si(9)-O(12)	109.1(4)	C(1)-C(6)-H(5)	119.1
O(13)-Si(9)-C(49B)	112.2(6)	C(5)-C(6)-H(5)	119.1
O(12)-Si(9)-C(49B)	108.8(6)	C(8A)-C(7A)-C(12A)	115(2)
O(13)-Si(9)-C(55B)	107.8(8)	C(8A)-C(7A)-Si(2)	127.3(19)
O(12)-Si(9)-C(55B)	110.8(8)	C(12A)-C(7A)-Si(2)	117.5(18)
C(49B)-Si(9)-C(55B)	108.1(11)	C(7A)-C(8A)-C(9A)	123.0(19)
C(55A)-Si(9)-C(49A)	117.3(15)	C(7A)-C(8A)-H(31)	118.5
O(13)-Si(9)-C(49A)	97.6(13)	C(9A)-C(8A)-H(31)	118.5
O(12)-Si(9)-C(49A)	102.9(9)	C(10A)-C(9A)-C(8A)	119.7(18)
Si(10)-O(13)-Si(9)	165.1(5)	C(10A)-C(9A)-H(32)	120.1
O(13)-Si(10)-O(14)	107.7(4)	C(8A)-C(9A)-H(32)	120.1
O(13)-Si(10)-C(61)	109.0(4)	C(9A)-C(10A)-C(11A)	124(2)
O(14)-Si(10)-C(61)	109.6(4)	C(9A)-C(10A)-H(33)	117.8
O(13)-Si(10)-C(67)	109.0(5)	C(11A)-C(10A)-H(33)	117.8
O(14)-Si(10)-C(67)	108.9(4)	C(10A)-C(11A)-C(12A)	116(2)
C(61)-Si(10)-C(67)	112.5(4)	C(10A)-C(11A)-H(34)	121.9
Si(5)-O(14)-Si(10)	165.2(5)	C(12A)-C(11A)-H(34)	121.9
C(2)-C(1)-C(6)	114.5(14)	C(7A)-C(12A)-C(11A)	121.2(19)
C(2)-C(1)-Si(1)	122.5(10)	C(7A)-C(12A)-H(35)	119.4
C(6)-C(1)-Si(1)	122.1(11)	C(11A)-C(12A)-H(35)	119.4
C(1)-C(2)-C(3)	123.3(17)	C(12B)-C(7B)-C(8B)	113(3)
C(1)-C(2)-H(1)	118.3	C(12B)-C(7B)-Si(2)	121(3)
C(3)-C(2)-H(1)	118.3	C(8B)-C(7B)-Si(2)	126(3)
C(4)-C(3)-C(2)	119.0(19)	C(7B)-C(8B)-C(9B)	127(3)
C(4)-C(3)-H(2)	120.5	C(7B)-C(8B)-H(41)	116.4
C(2)-C(3)-H(2)	120.5	C(9B)-C(8B)-H(41)	116.4
C(5)-C(4)-C(3)	124(2)	C(10B)-C(9B)-C(8B)	113(3)
C(5)-C(4)-H(3)	117.8	C(10B)-C(9B)-H(42)	123.3
C(3)-C(4)-H(3)	117.8	C(8B)-C(9B)-H(42)	123.3

C(9B)-C(10B)-C(11B)	127(4)	C(20A)-C(21A)-C(22A)	119(2)
C(9B)-C(10B)-H(43)	116.7	C(20A)-C(21A)-H(47)	120.6
C(11B)-C(10B)-H(43)	116.7	C(22A)-C(21A)-H(47)	120.6
C(10B)-C(11B)-C(12B)	113(3)	C(21A)-C(22A)-C(23A)	121(2)
C(10B)-C(11B)-H(44)	123.5	C(21A)-C(22A)-H(48)	119.4
C(12B)-C(11B)-H(44)	123.5	C(23A)-C(22A)-H(48)	119.4
C(7B)-C(12B)-C(11B)	126(3)	C(24A)-C(23A)-C(22A)	119(2)
C(7B)-C(12B)-H(45)	116.9	C(24A)-C(23A)-H(49)	120.7
C(11B)-C(12B)-H(45)	116.9	C(22A)-C(23A)-H(49)	120.7
C(14)-C(13)-C(18)	115.3(9)	C(19A)-C(24A)-C(23A)	121(2)
C(14)-C(13)-Si(3)	122.6(8)	C(19A)-C(24A)-H(50)	119.5
C(18)-C(13)-Si(3)	122.0(8)	C(23A)-C(24A)-H(50)	119.5
C(13)-C(14)-C(15)	122.2(11)	C(24B)-C(19B)-C(20B)	114(3)
C(13)-C(14)-H(6)	118.9	C(24B)-C(19B)-Si(4)	123(3)
C(15)-C(14)-H(6)	118.9	C(20B)-C(19B)-Si(4)	122(3)
C(16)-C(15)-C(14)	119.6(12)	C(21B)-C(20B)-C(19B)	126(3)
C(16)-C(15)-H(7)	120.2	C(21B)-C(20B)-Cl(2B)	81(2)
C(14)-C(15)-H(7)	120.2	C(19B)-C(20B)-Cl(2B)	153(3)
C(17)-C(16)-C(15)	121.2(12)	C(21B)-C(20B)-H(51)	116.9
C(17)-C(16)-H(8)	119.4	C(19B)-C(20B)-H(51)	116.9
C(15)-C(16)-H(8)	119.4	Cl(2B)-C(20B)-H(51)	36.1
C(16)-C(17)-C(18)	118.3(13)	C(22B)-C(21B)-C(20B)	114(3)
C(16)-C(17)-H(9)	120.9	C(22B)-C(21B)-Cl(2B)	174(3)
C(18)-C(17)-H(9)	120.9	C(20B)-C(21B)-Cl(2B)	59(2)
C(13)-C(18)-C(17)	123.0(12)	C(22B)-C(21B)-H(52)	122.8
C(13)-C(18)-H(10)	118.5	C(20B)-C(21B)-H(52)	122.8
C(17)-C(18)-H(10)	118.5	Cl(2B)-C(21B)-H(52)	63.4
C(24A)-C(19A)-C(20A)	118(2)	C(21B)-C(22B)-C(23B)	126(3)
C(24A)-C(19A)-Si(4)	121.6(18)	C(21B)-C(22B)-H(53)	117.2
C(20A)-C(19A)-Si(4)	120.1(17)	C(23B)-C(22B)-H(53)	117.2
C(21A)-C(20A)-C(19A)	122(2)	C(22B)-C(23B)-C(24B)	114(3)
C(21A)-C(20A)-H(46)	119.0	C(22B)-C(23B)-H(54)	123.0
C(19A)-C(20A)-H(46)	119.0	C(24B)-C(23B)-H(54)	123.0

C(19B)-C(24B)-C(23B)	126(3)
C(19B)-C(24B)-H(55)	117.1
C(23B)-C(24B)-H(55)	117.1
C(24C)-C(19C)-C(20C)	117(2)
C(24C)-C(19C)-Si(4)	123(2)
C(20C)-C(19C)-Si(4)	120(2)
C(21C)-C(20C)-C(19C)	122(2)
C(21C)-C(20C)-H(56)	118.9
C(19C)-C(20C)-H(56)	118.9
C(22C)-C(21C)-C(20C)	117(2)
C(22C)-C(21C)-H(57)	121.4
C(20C)-C(21C)-H(57)	121.4
C(23C)-C(22C)-C(21C)	123(3)
C(23C)-C(22C)-H(58)	118.4
C(21C)-C(22C)-H(58)	118.4
C(22C)-C(23C)-C(24C)	117(3)
C(22C)-C(23C)-H(59)	121.3
C(24C)-C(23C)-H(59)	121.3
C(23C)-C(24C)-C(19C)	123(2)
C(23C)-C(24C)-H(60)	118.6
C(19C)-C(24C)-H(60)	118.6
C(30A)-C(25A)-C(26A)	120(2)
C(30A)-C(25A)-Si(5)	118.9(19)
C(26A)-C(25A)-Si(5)	121(2)
C(25A)-C(26A)-C(27A)	120(3)
C(25A)-C(26A)-H(91)	120.0
C(27A)-C(26A)-H(91)	120.0
C(28A)-C(27A)-C(26A)	120(3)
C(28A)-C(27A)-H(92)	119.8
C(26A)-C(27A)-H(92)	119.8
C(29A)-C(28A)-C(27A)	119(2)
C(29A)-C(28A)-H(93)	120.4
C(27A)-C(28A)-H(93)	120.4
C(28A)-C(29A)-C(30A)	120(2)
C(28A)-C(29A)-H(94)	119.7
C(30A)-C(29A)-H(94)	119.8
C(25A)-C(30A)-C(29A)	120(2)
C(25A)-C(30A)-H(95)	120.0
C(29A)-C(30A)-H(95)	120.0
C(30B)-C(25B)-C(26B)	118.4(18)
C(30B)-C(25B)-Si(5)	123.6(12)
C(26B)-C(25B)-Si(5)	118.0(17)
C(27B)-C(26B)-C(25B)	115(3)
C(27B)-C(26B)-H(86)	122.4
C(25B)-C(26B)-H(86)	122.4
C(28B)-C(27B)-C(26B)	127(2)
C(28B)-C(27B)-H(87)	116.7
C(26B)-C(27B)-H(87)	116.7
C(27B)-C(28B)-C(29B)	116(2)
C(27B)-C(28B)-H(88)	121.9
C(29B)-C(28B)-H(88)	121.9
C(28B)-C(29B)-C(30B)	122.2(19)
C(28B)-C(29B)-H(89)	118.9
C(30B)-C(29B)-H(89)	118.9
C(29B)-C(30B)-C(25B)	121.1(16)
C(29B)-C(30B)-H(90)	119.5
C(25B)-C(30B)-H(90)	119.5
C(36)-C(31)-C(32)	116.9(9)
C(36)-C(31)-Si(6)	122.8(8)
C(32)-C(31)-Si(6)	120.3(8)
C(33)-C(32)-C(31)	119.4(10)
C(33)-C(32)-H(11)	120.3
C(31)-C(32)-H(11)	120.3
C(34)-C(33)-C(32)	123.7(11)
C(34)-C(33)-H(12)	118.2
C(32)-C(33)-H(12)	118.2

C(33)-C(34)-C(35)	118.1(11)	C(40B)-C(39B)-C(38B)	114(3)
C(33)-C(34)-H(13)	120.9	C(40B)-C(39B)-H(82)	122.9
C(35)-C(34)-H(13)	120.9	C(38B)-C(39B)-H(82)	122.9
C(34)-C(35)-C(36)	120.1(12)	C(41B)-C(40B)-C(39B)	126(3)
C(34)-C(35)-H(14)	120.0	C(41B)-C(40B)-H(83)	117.1
C(36)-C(35)-H(14)	120.0	C(39B)-C(40B)-H(83)	117.1
C(31)-C(36)-C(35)	121.8(11)	C(40B)-C(41B)-C(42B)	115(3)
C(31)-C(36)-H(15)	119.1	C(40B)-C(41B)-H(84)	122.6
C(35)-C(36)-H(15)	119.1	C(42B)-C(41B)-H(84)	122.6
C(42A)-C(37A)-C(38A)	117.0(15)	C(37B)-C(42B)-C(41B)	125(3)
C(42A)-C(37A)-Si(7)	121.8(15)	C(37B)-C(42B)-H(85)	117.6
C(38A)-C(37A)-Si(7)	121.2(13)	C(41B)-C(42B)-H(85)	117.6
C(39A)-C(38A)-C(37A)	120.3(15)	C(48)-C(43)-C(44)	116.3(9)
C(39A)-C(38A)-H(36)	119.8	C(48)-C(43)-Si(8)	122.1(7)
C(37A)-C(38A)-H(36)	119.8	C(44)-C(43)-Si(8)	121.6(8)
C(40A)-C(39A)-C(38A)	119.1(18)	C(43)-C(44)-C(45)	121.5(11)
C(40A)-C(39A)-H(37)	120.5	C(43)-C(44)-H(16)	119.3
C(38A)-C(39A)-H(37)	120.5	C(45)-C(44)-H(16)	119.3
C(41A)-C(40A)-C(39A)	120.6(19)	C(46)-C(45)-C(44)	120.0(11)
C(41A)-C(40A)-H(38)	119.7	C(46)-C(45)-H(17)	120.0
C(39A)-C(40A)-H(38)	119.7	C(44)-C(45)-H(17)	120.0
C(40A)-C(41A)-C(42A)	122.0(17)	C(45)-C(46)-C(47)	119.7(10)
C(40A)-C(41A)-H(39)	119.0	C(45)-C(46)-H(18)	120.1
C(42A)-C(41A)-H(39)	119.0	C(47)-C(46)-H(18)	120.1
C(37A)-C(42A)-C(41A)	120.4(17)	C(48)-C(47)-C(46)	119.0(11)
C(37A)-C(42A)-H(40)	119.8	C(48)-C(47)-H(19)	120.5
C(41A)-C(42A)-H(40)	119.8	C(46)-C(47)-H(19)	120.5
C(38B)-C(37B)-C(42B)	115(3)	C(47)-C(48)-C(43)	123.5(10)
C(38B)-C(37B)-Si(7)	125(3)	C(47)-C(48)-H(20)	118.3
C(42B)-C(37B)-Si(7)	120(3)	C(43)-C(48)-H(20)	118.3
C(37B)-C(38B)-C(39B)	126(4)	C(54A)-C(49A)-C(50A)	119(3)
C(37B)-C(38B)-H(81)	117.1	C(54A)-C(49A)-Si(9)	120(2)
C(39B)-C(38B)-H(81)	117.1	C(50A)-C(49A)-Si(9)	121(2)

C(49A)-C(50A)-C(51A)	121(3)
C(49A)-C(50A)-H(61)	119.3
C(51A)-C(50A)-H(61)	119.3
C(50A)-C(51A)-C(52A)	119(3)
C(50A)-C(51A)-H(62)	120.7
C(52A)-C(51A)-H(62)	120.7
C(51A)-C(52A)-C(53A)	121(3)
C(51A)-C(52A)-H(63)	119.3
C(53A)-C(52A)-H(63)	119.3
C(54A)-C(53A)-C(52A)	118(3)
C(54A)-C(53A)-H(64)	120.8
C(52A)-C(53A)-H(64)	120.8
C(49A)-C(54A)-C(53A)	121(3)
C(49A)-C(54A)-H(65)	119.3
C(53A)-C(54A)-H(65)	119.3
C(50B)-C(49B)-C(54B)	113.8(19)
C(50B)-C(49B)-Si(9)	122.8(17)
C(54B)-C(49B)-Si(9)	123.4(16)
C(49B)-C(50B)-C(51B)	125(2)
C(49B)-C(50B)-H(66)	117.6
C(51B)-C(50B)-H(66)	117.6
C(52B)-C(51B)-C(50B)	118(2)
C(52B)-C(51B)-H(67)	120.9
C(50B)-C(51B)-H(67)	120.9
C(51B)-C(52B)-C(53B)	119.1(19)
C(51B)-C(52B)-H(68)	120.4
C(53B)-C(52B)-H(68)	120.4
C(52B)-C(53B)-C(54B)	122(2)
C(52B)-C(53B)-H(69)	118.9
C(54B)-C(53B)-H(69)	118.9
C(49B)-C(54B)-C(53B)	121(2)
C(49B)-C(54B)-H(70)	119.4
C(53B)-C(54B)-H(70)	119.4
C(60A)-C(55A)-C(56A)	119(3)
C(60A)-C(55A)-Si(9)	123(2)
C(56A)-C(55A)-Si(9)	118.7(19)
C(55A)-C(56A)-C(57A)	122(3)
C(55A)-C(56A)-H(71)	119.1
C(57A)-C(56A)-H(71)	119.1
C(58A)-C(57A)-C(56A)	118(3)
C(58A)-C(57A)-H(72)	121.0
C(56A)-C(57A)-H(72)	121.0
C(57A)-C(58A)-C(59A)	122(3)
C(57A)-C(58A)-H(73)	119.2
C(59A)-C(58A)-H(73)	119.2
C(60A)-C(59A)-C(58A)	118(3)
C(60A)-C(59A)-H(74)	120.9
C(58A)-C(59A)-H(74)	120.9
C(55A)-C(60A)-C(59A)	122(3)
C(55A)-C(60A)-H(75)	119.2
C(59A)-C(60A)-H(75)	119.2
C(60B)-C(55B)-C(56B)	113(2)
C(60B)-C(55B)-Si(9)	123(2)
C(56B)-C(55B)-Si(9)	123.4(18)
C(55B)-C(56B)-C(57B)	127(2)
C(55B)-C(56B)-H(76)	116.6
C(57B)-C(56B)-H(76)	116.6
C(56B)-C(57B)-C(58B)	115(2)
C(56B)-C(57B)-H(77)	122.3
C(58B)-C(57B)-H(77)	122.3
C(59B)-C(58B)-C(57B)	121(2)
C(59B)-C(58B)-H(78)	119.4
C(57B)-C(58B)-H(78)	119.4
C(58B)-C(59B)-C(60B)	120(2)
C(58B)-C(59B)-H(79)	120.1
C(60B)-C(59B)-H(79)	120.1

C(55B)-C(60B)-C(59B)	124(2)
C(55B)-C(60B)-H(80)	118.2
C(59B)-C(60B)-H(80)	118.2
C(66)-C(61)-C(62)	115.3(10)
C(66)-C(61)-Si(10)	122.8(8)
C(62)-C(61)-Si(10)	121.7(9)
C(63)-C(62)-C(61)	123.5(13)
C(63)-C(62)-H(21)	118.2
C(61)-C(62)-H(21)	118.2
C(62)-C(63)-C(64)	120.3(14)
C(62)-C(63)-H(22)	119.8
C(64)-C(63)-H(22)	119.8
C(63)-C(64)-C(65)	119.2(13)
C(63)-C(64)-H(23)	120.4
C(65)-C(64)-H(23)	120.4
C(64)-C(65)-C(66)	118.4(13)
C(64)-C(65)-H(24)	120.8
C(66)-C(65)-H(24)	120.8
C(61)-C(66)-C(65)	123.2(11)
C(61)-C(66)-H(25)	118.4
C(65)-C(66)-H(25)	118.4
C(68)-C(67)-C(72)	117.4(10)
C(68)-C(67)-Si(10)	119.7(9)
C(72)-C(67)-Si(10)	122.6(8)
C(67)-C(68)-C(69)	118.1(15)
C(67)-C(68)-H(26)	120.9
C(69)-C(68)-H(26)	120.9
C(70)-C(69)-C(68)	124.0(17)
C(70)-C(69)-H(27)	118.0
C(68)-C(69)-H(27)	118.0
C(71)-C(70)-C(69)	117.0(17)
C(71)-C(70)-H(28)	121.5
C(69)-C(70)-H(28)	121.5
C(70)-C(71)-C(72)	119.5(14)
C(70)-C(71)-H(29)	120.2
C(72)-C(71)-H(29)	120.2
C(71)-C(72)-C(67)	123.1(12)
C(71)-C(72)-H(30)	118.4
C(67)-C(72)-H(30)	118.4
Cl(2A)-C(73A)-Cl(1A)	101(4)
Cl(2A)-C(73A)-H(96)	111.5
Cl(1A)-C(73A)-H(96)	111.5
Cl(2A)-C(73A)-H(97)	111.5
Cl(1A)-C(73A)-H(97)	111.5
H(96)-C(73A)-H(97)	109.3
Cl(2B)-C(73B)-Cl(1B)	106(4)
Cl(2B)-C(73B)-H(98)	110.5
Cl(1B)-C(73B)-H(98)	110.5
Cl(2B)-C(73B)-H(99)	110.5
Cl(1B)-C(73B)-H(99)	110.5
H(98)-C(73B)-H(99)	108.7
C(73B)-Cl(2B)-C(20B)	139(2)
C(73B)-Cl(2B)-C(21B)	102(2)
C(20B)-Cl(2B)-C(21B)	39.8(9)

Symmetry transformations used to generate equivalent atoms:
